# Infodemic: the effect of death-related thoughts on news-sharing

**DOI:** 10.1186/s41235-021-00306-0

**Published:** 2021-05-20

**Authors:** Amy J. Lim, Edison Tan, Tania Lim

**Affiliations:** 1 Murdoch Singapore, Murdoch University Singapore, Kings Centre #03-01, 390 Havelock Road, Singapore, 169662 Singapore; 2 School of Social Sciences, Singapore Management University, 90 Stamford Road, Level 4, Singapore, 178903 Singapore

**Keywords:** Fake news, Terror management theory, Mortality salience, Death-thought accessibility, News-sharing

## Abstract

Research on the sharing of fake news has primarily focused on the manner in which fake news spreads and the literary style of fake news. These studies, however, do not explain how characteristics of fake news could affect people’s inclination toward sharing these news articles. Drawing on the Terror Management Theory, we proposed that fake news is more likely to elicit death-related thoughts than real news. Consequently, to manage the existential anxiety that had been produced, people share the news articles to feel connected to close others as a way of resolving the existential anxiety. Across three experimental studies (total *N* = 416), we found that it was not news type per se (i.e., real versus fake news) that influenced news-sharing intentions; instead, it was the increased accessibility to death-related thoughts elicited from the content of news articles that motivated news-sharing. The findings support the Terror Management framework and contribute to the existing literature by providing an empirical examination of the underlying psychological motive behind fake news-sharing tendencies.

**Supplementary information:**

The online version contains supplementary material available at 10.1186/s41235-021-00306-0.

## Significance Statement

The poor veracity of information proliferating on social media has not only misled readers but it also triggers behaviors that can potentially harm others. Research on the sharing of fake news, to date, has primarily focused on the manner in which fake news spreads and the literary style of fake news. Such findings do not explain *why* people are inclined to share fake news. Drawing on the Terror Management Theory, we elucidate the underlying factor behind news-sharing. Specifically, we proposed that death-related thoughts elicited from fake news articles motivate news-sharing. In three experimental studies, we demonstrated that it was not news type per se (i.e., fake news or real news) that influenced news-sharing inclinations; instead, it was death-related thoughts elicited from the content of news articles that motivated news-sharing. The findings of this paper contribute to the existing literature by providing an empirical examination of the underlying psychological motive behind fake news-sharing tendencies. The findings of this paper can also inform researchers, practitioners and regulatory bodies in designing effective interventions to manage the spread of fake news.

## Background

Fake news, broadly defined as contents presented in news formats that lacks facticity, is intended to mislead readers (Allcott & Gentzkow, 2017; Egelhofer & Lecheler, 2019; Tandoc et al., 2018), can clearly—and unnecessarily—set off behaviors with real consequences. For example, a gunman opened fire at a pizzeria believing it was the site of an underground child sex ring (Lopez, 2016), while Pakistan’s Defence Minster directed a nuclear threat to Israel in response to a made-up story on social media (Goldman, 2016). Fake news is a problem not only of people taking in wrong information but also of people being confused about facts of current issues and events (Barthel et al., 2016)); for instance, the controversy around the effectiveness of hydroxychloroquine and chloroquine in treating the Coronavirus Disease 2019 (COVID-19; Liu et al., 2020). Furthermore, these phenomena have demonstrated some level of resistance to corrective intervention. When presented with corrected information after reading fake news, people’s adjusted attitudes remained biased (De Keersmaecker & Roets, 2017); worse yet, people reinforced their belief in the initial yet incorrect information (Nyhan & Reifler, 2010; Thorson, 2016). These studies demonstrate the persistence of misinformation, and more critically, highlight the pernicious nature of fake news.

Recent communication developments, especially social media platforms, exacerbate the problems with fake news as it allows false information to be planted and spread far more easily than before (Bessi, 2017). Social bots, fake accounts that post content and interact with users, often take the blame for the spread of fake news (Bessi & Ferrara, 2016), which resulted in a call for interventions aimed at curtailing bots (Shao et al., 2018; Yang et al., 2019). However, this proved to be a misdirected effort given that the spread of fake news is still evident even after accounting for bot activity, suggesting that fake news is propagated by humans equally, if not more than, social bots (Vosoughi et al., 2018). As such, there is a need for research to focus on understanding the factors that influence people to share and propagate fake news.

### Sharing fake news

Studies examining the spread of fake news have largely focused on the manner in which it spreads (e.g., Metaxas & Mustafaraj, 2010; Mustafaraj & Metaxas, 2017) and the characteristics of fake news that encourage its sharing (for a review, see Zhou & Zafarani, 2020). For example, fake news spreads by having fictitious user accounts infiltrate into a community of social media users who are engaged in conversations on a particular topic; these accounts then hijack discussions with fake news, which then spread organically via sharing and are consequently disseminated into the extended networks of users (Mustafaraj & Metaxas, 2017). Researchers also found that fake news tends to resurface more often than real news (Shin et al., 2018), which suggests that fake news tends to be shared more than real news. Studies have identified literary characteristics of fake news that encourage the sharing of such articles. For instance, fake news contains more novelty, which captures the attention of people (Vosoughi et al., 2018). Additionally, fake news also contains longer titles, is written simplistically and contains repetitive content (Horne & Adali, 2017); these features make it easier for people to understand the articles (Schwarz et al., 2016).

While these studies elucidate *how* fake news spreads and *what* makes fake news more likely to be shared than real news, they do not explain *why* people tend to share fake news over real news. In other words, the underlying psychological mechanism behind the inclination to share fake news articles remains unclear.

### Terror management theory

To investigate this, we employed the Terror Management Theory, a framework based on social evolutionary psychology that addresses phenomena related to behaviors aimed at resolving existential anxiety (Becker, 1973; Greenberg et al., 1986; Pyszczynski et al., 2015). The Terror Management Theory (TMT; Becker, 1973; Greenberg et al., 1986; Pyszczynski et al., 2015) posits that the awareness of the inevitability of death produces existential anxiety for humans who possess an inherent proclivity for self-preservation. Existential anxiety is managed by an anxiety-buffering system consisting of defenses focused on cultural worldviews, self-esteem, and close relationships (Pyszczynski et al., 2020). Cultural worldviews are shared beliefs about life, standards for valued behavior, and the promise of literal immortality (the continuation of life after death such as heaven) and symbolic immortality (the continuation of one’s contribution to something greater than oneself after death such as a family) for living up to the standards. Self-esteem refers to the sense of personal value from believing that one has lived up to the standards of their cultural worldview. Although initial work on TMT has focused on cultural worldviews and self-esteem as coping mechanisms, more recent lines of research have proposed that people also manage existential anxiety by investing in close relationships (for a review, see Mikulincer et al., 2004). Close relationships, on top of validating one’s worldviews and self-esteem, provide a sense of belonging and comfort (Mikulincer et al., 2003). These defenses provide the sense that there is meaning to one’s life (for a review, see Pyszczynski et al., 2015). In essence, these defenses manage existential anxiety, produced by thoughts of death, by reducing the accessibility to these thoughts (Arndt et al. 1997).

#### Application of TMT to fake news

The TMT framework has been employed in various works relating to media psychology. Notably, this framework has been used to explain Americans’ nationalistic responses following the 9/11 attacks (Pyszczynski et al., 2003) and the hostile sentiments against alternative values and views following the Berlin Christmas Market attack (Fischer-Preßler et al., 2019). These examples demonstrate that news information can invoke terror management defenses, which lends confidence to the applicability of the TMT framework to understand people’s responses to fake news.

Anecdotal evidence suggests that fake news is more likely than real news in eliciting, and thus increasing the accessibility to, death-related thoughts. Fake news tends to be written to arouse emotions, particularly negative ones (Pérez-Rosas et al., 2018). Vosoughi et al. (2018) compared people’s responses to fake news with real news and found that people responded to fake news with fear, disgust and surprise more so than real news. More critically, fake news tends to be associated with death-related themes. For instance, the array of false death announcements of public figures on social media (Nansen et al., 2019), fake news regarding an Ebola outbreak in Atlanta that was allegedly used by Russian operatives to sow discord in the USA (Lysenko & Brooks, 2018), and the alleged discovery of weapons of mass destruction in Iraq (Kull et al., 2003). Fake news consistently used sensational words such as “*accident*” or “*death*” (Molina et al., 2019). As such, while any news that contains death-related information would elicit and increase the accessibility to death-related thoughts, the way fake news is produced to create sensation and include death-related themes makes fake news more likely to elicit death-related thoughts than real news for any given content. Consequently, it is likely that fake news generates existential anxiety more than real news.

Accordingly, as per TMT, terror management defenses are activated upon the increased accessibility to death-related thoughts to resolve the existential anxiety that has been produced. Thus, people are motivated to forestall the threat of death and convince themselves that they lead meaningful lives by affirming their worldviews, enhancing their self-esteem, and seeking close relationships. Findings from research on news-sharing suggest that sharing news articles is a manifestation of the anxiety-buffering system-it is a form of seeking close relationships. Sharing news articles was found to provide a sense of belonging and it allowed one to feel more connected to others (Quinn & Oldmeadow, 2013; Ryan et al., 2017; van Eldik et al., 2019). Moreover, intentions to share news articles on social media were higher when one perceived their social ties within their social media network to be strong (Ma et al., 2014). These studies suggest that as much as sharing news articles made one feel closer to others, people share news articles to feel even closer to others to *whom they are close to*. This supports the idea that the practice of sharing news articles buffers existential anxiety by providing a sense of connectedness to one’s close relationships.

With fake news more likely than real news to elicit death-related thoughts and produce existential anxiety, it is likely that fake news activates terror management defenses more than real news to mitigate the resulting existential anxiety. And given that sharing news articles mitigates existential anxiety by allowing one to feel closer to their close relationships, it is more likely for people to share fake news articles than real news articles. In other words, the underlying psychological mechanism behind the sharing of fake news lies with its accessibility to death-related thoughts and the production of existential anxiety: fake news articles induce death-related thoughts and existential anxiety, consequently, sharing these articles helps to reduce accessibility to death-related thoughts and resolves the existential anxiety produced. Research demonstrating the tendency of people to share news information associated with negative feelings (e.g., worry and fear), compared to information associated with positive feelings (e.g., relieve) and neutral feelings (e.g., indifference) (Chen & Sakamoto, 2013), provides initial evidence that sharing news articles can alleviate the negative state people find themselves in when exposed to negative news.

## The present research

Work investigating the characteristics of fake news content (e.g., Vosoughi et al., 2018) provides a guide to understanding the appeals of fake news. It is evident that fake news is crafted in manners to achieve virality (Vosoughi et al., 2018), often by means of triggering affective processes that motivate news-sharing intention (Bakir & McStay, 2018). However, while these studies elucidate the *whats* of fake news, they do not explain the *why*—why would these characteristics of fake news cause people to want to share them with others?

Employing the TMT framework, this paper proposes that the increased accessibility to death-related thoughts is the unconscious driving force behind news-sharing. Since fake news is crafted to instigate death-related thoughts, which produces existential anxiety, and according to TMT, people react to this by investing in close relationships, we propose that people share these pieces of (mis)information to reduce the accessibility to death-related thoughts and resolve existential anxiety that had been produced.

Through three experiments, we investigated: (1) if fake news elicits death-related thoughts more than real news, and (2) if death-related thoughts motivate news-sharing intent. In doing so, we hope to shed light on the underlying mechanism responsible for fake news-sharing behaviors.

## Study 1

Research has shown that fake news tends to be emotionally charged (Pérez-Rosas et al., 2018), evokes negative emotions (Vosoughi et al., 2018) and contains death-related themes (Molina et al., 2019). These studies suggest that fake news is more likely to elicit and increase the accessibility to death-related thoughts compared to real news. As such, the aim of Study 1 is to investigate if fake news elicits death-related thoughts more than real news.

### Participants

A total of 97 participants were recruited from Amazon’s Mechanical Turk (46 females, *M*_age_ = 34.99, *SD*_age_ = 5.59). All participants resided in the USA; most of them were Americans (95 Americans) and identified “Caucasian” as their ethnic identity (74 Caucasians, 7 African Americans, 2 Hispanics, 7 Asians, 5 Native Americans, 2 N/A). All but 1 participant indicated that their native language was English.

### Design and procedure

This study consisted of two between-participant conditions: fake news condition and real news condition (*N*_fake news_ = 50; *N*_real news_ = 47). After providing informed consent, participants were randomly assigned to one of the two conditions where they were asked to read either a series of 8 fake news articles (fake news condition) or a series of 8 real news articles (real news condition). Next, participants responded to items assessing how they felt and items assessing death-thought accessibility after reading the news articles. Finally, they provided demographic information before completing the study.

### Materials

#### News articles

Social media, such as Facebook, are now commonly used as platforms for people to consume news (Fletcher & Nielsen, 2018; Winter et al., 2015); as such, this study employed Facebook posts as the stimuli. Participants in the fake news condition were shown a series of 8 Facebook posts containing fake news, which were sourced and randomly selected from reputable independent fact-checkers (e.g., Black Dot Research, Snopes). In contrast, participants in the real news condition were shown a series of 8 Facebook posts containing real news, which were sourced and randomly selected from reputable media outlets (e.g., The Guardian, Reuters, Channel News Asia). The series of Facebook posts for both fake and real news conditions were mock-ups created based on the headlines of selected news stories and summaries of their content (see Additional file 1: Appendix 1).

#### Affect

The 20-item Positive and Negative Affect Schedule (PANAS; Watson et al., 1988) was used to measure the extent to which participants experienced negative emotions (e.g., *distressed, upset, scared, afraid*) and positive emotions (e.g., *excited, proud, enthusiastic, inspired*) after reading the series of news articles. They were instructed to indicate how they felt at that point of time after reading the news articles on a 5-point Likert scale (1 = *not at all*, 5 = *extremely*). Items were averaged to form an index for negative affect (*M* = 1.87, *SD* = 0.93, α = 0.95) and positive affect (*M* = 2.70, *SD* = 0.94, α = 0.91).

#### Death-thought accessibility

Death-thought accessibility was assessed through two items: participants indicated the extent to which they felt “a sense of threat” and “death awareness” on a 5-point Likert scale (1 = *not at all*, 5 = *extremely*). The two items were correlated (*r* = 0.76) and were thus averaged to form a composite score for death-thought accessibility (*M* = 1.97, *SD* = 1.03).

### Results and discussion

An independent samples t-test was conducted to examine the mean differences in negative affect, positive affect and death-thought accessibility elicited by fake news articles versus real news articles. Results revealed that death-thought accessibility was higher in the real news condition (*M* = 2.21, *SD* = 1.06) than the fake news condition (*M* = 1.75, *SD* = 0.96), *t*(95) = 2.25, *p* = 0.026, 95% CI [0.06, 0.87]; *d* = 0.46. A sensitivity analysis using G-Power indicated that given a total sample size of 97 (*N*_fake news_ = 50; *N*_real news_ = 47) and at α = 0.05, the minimum effect size to detect a power of 0.80 is 0.58. Hence, the effect observed in this study should be interpreted with caution. The sensitivity analysis also indicated a sample size of 150 participants would be required to observe the effect size of 0.46, which was found for this study, with power of 0.80 and at α = 0.05. Results also showed that negative affect was higher in the real news condition (*M* = 2.06, *SD* = 1.03) than the fake news condition (*M* = 1.69, *SD* = 0.78), though this difference approached marginal significance *t*(85.76) = 1.99, *p* = 0.049, 95% CI [0.001, 0.74], *d* = 0.41. Fake news articles and real news articles elicited similar levels for positive affect (*p* = 0.28).

We conducted additional tests to check that these results were due to the unique effect of news type (fake versus real news) and were not conflated with the effects that may be present due to demographic characteristics (i.e., age, gender, nationality, ethnic identity, and native language). These tests included separate simple linear regressions to determine the demographic characteristics that may have an effect on death-thought accessibility, negative affect, and positive affect. This was followed by a series of ANCOVAs with the demographic characteristics that had a significant effect on death-thought accessibility, negative affect, and positive affect included as covariates. Results indicated that even when demographic variables were accounted for, the initial findings remained—death-thought accessibility and negative affect were higher in the real news condition than the fake news condition, while positive affect was similar across the two conditions.

Taken together, contrary to our predictions, the findings showed that *real* news elicited *higher* levels of death-thought accessibility and negative affect compared to fake news. Fake news and real news elicited similar levels of positive affect.

## Study 2

Study 2 served to replicate our findings in Study 1 that real news increased death-thought accessibility more than fake news. Additionally, Study 2 examined if increased death-thought accessibility, elicited through the news articles, would influence one’s attention to death-related cues and news-sharing intentions.

### Participants

Given the effect sizes we have found for death-thought accessibility and negative affect in Study 1, we adopted a more conservative approach in our a-priori power analysis using G-Power (Faul & Erdfelder, 1992). Based on the smaller effect size of *d* = 0.41 that we have obtained in Study 1, a power of 0.80 and a t-test between means at *α* = 0.05, the minimum sample size of *n* = 190 (*n* = 95 per condition) is required to detect such an effect. We recruited 195 participants through Amazon’s Mechanical Turk (103 females, *M*_age_ = 34.04, *SD*_age_ = 6.25). All participants resided in the USA; most of them were Americans (186 Americans) and identified “Caucasian” as their ethnic identity (138 Caucasians, 19 African Americans, 6 Hispanics, 15 Asians, 8 Native Americans, 9 N/A). All but 1 participant indicated that their native language was English.

### Design and procedure

The procedure in Study 2 is similar to that of Study 1. Participants were randomly assigned to one of two conditions in a between-participants design (*N*_fake news_ = 101, *N*_real news_ = 94). After providing informed consent, participants were assigned to one of the two conditions, where they were asked to read either a series of 8 fake news articles (i.e., fake news condition) or a series of 8 real news articles (i.e., real news condition). After which, they indicated how they felt after and responded to items that measured death-thought accessibility reading the news articles. They then completed a 20-pair question-word task before responding to questions that assessed news-sharing intention. Lastly, they provided their demographic information before completing the survey.

### Materials

#### News articles

The series of fake and real news articles used in this study were the same ones that are employed in Study 1. Participants in the fake news condition were shown the series of 8 Facebook posts that contained fake news, while participants in the real news condition were shown the series of 8 Facebook posts that contained real news.

#### Affect

We assessed negative and positive affect using the same instrument from Study 1. Similarly, items were averaged to form a single index for negative affect (*M* = 1.75, *SD* = 0.95, α = 0.96) and positive affect (*M* = 2.80, *SD* = 0.95, α = 0.92).

#### Death-thought accessibility

We assessed death-thought accessibility using the same items from Study 1. The two items were correlated (*r* = 0.82) and hence were averaged to form a single index for death-thought accessibility (*M* = 1.87, *SD* = 1.17).

#### Attention to death-related cues

Attentiveness to death-related cues was assessed through a question-word pair task modified from Rogers et al. (1977) to capture the selective encoding of death-related words. In this task, participants were shown a word and asked if it contained a specific letter. For example, the screen displayed the question “Does the following word contain the letter I”? along with the word “Coffin” for 4 s. Participants were required to respond as quickly and as accurately as they can before the 4 s were up. Participants were exposed to 20 of such trials, 10 of these trials contained death-related words (*coffin, extinct, tomb, grave*) while the other 10 trials contained neutral words (*tissue, yellow, starfish, carton*). At the end of 20 trials, participants were asked to recall as many words as possible from the question-word pair. The number of death-related words they recalled served as an index for their attention to death-related cues (*M* = 1.65, *SD* = 1.51).

#### News-sharing intention

News-sharing intention was assessed with three items from Lee and Ma’s (2012) work on news-sharing in social media. Participants responded to the statements, “I intend to share news stories on Facebook in the future,” “I expect to share news stories contributed by other users,” and “I plan to share news stories on Facebook regularly.” We also included another item, “I am likely to share these news stories,” to measure the news-sharing intent specific to the news stories participants read in the study. Participants responded to these items on a 5-point Likert scale (1 = *strongly disagree,* 5 = *strongly agree*). The set of three items adopted from Lee and Ma (2012) correlated with the single item measure we included for this study (*r* = 0.84). Hence, items were averaged to form a single index for news-sharing intention (*M* = 2.44, *SD* = 1.27, α = 0.96).

### Results

#### Affect

Independent samples t-tests were conducted to examine the mean differences in negative affect and positive affect elicited by fake news articles versus real news articles. Results revealed that negative affect was higher in the real news condition (*M* = 1.90, *SD* = 1.01) than the fake news condition (*M* = 1.61, *SD* = 0.87), *t*(193) = 2.12, *p* = 0.04, 95% CI [0.02, 0.55]; *d* = 0.30. Positive affect was similar for both fake news condition and real news condition (*p* = 0.39).

#### Death-thought accessibility

An independent samples t-test was conducted to examine if death-thought accessibility was higher in the real news condition than the fake news condition, as we had observed in Study 1. Results revealed that death-thought accessibility was significantly higher in the real news condition (*M* = 2.12, *SD* = 1.26) than the fake news conditions (*M* = 1.63, *SD* = 1.03), *t*(180.17) = 2.98, *p* < 0.01, 95% CI [0.17, 0.82]; *d* = 0.43, consistent with the findings of Study 1.

#### Attention to death-related cues

Given the positive skew observed in the recall of death-related words (skewness = 1.58), the data were log-transformed for the analysis. An independent samples t-test was conducted to examine if attention to death-related cues differed across the real and fake news conditions. Results revealed that participants in both the real news condition and fake news condition recalled a similar number of death-related words (*p* = 0.42).

To explore if death-thought accessibility influenced participants’ attention to death-related cues, we conducted a mediation analysis using Hayes’ PROCESS model 4 (Hayes, 2018) where news type was included as the independent variable (coded as 0: real news articles, 1: fake news articles), death-thought accessibility was included as the mediator, and the number of death-related words recalled (log-transformed) was included as the dependent variable. As with our earlier findings, news type was negatively associated with death-thought accessibility, *B* =− 0.49, *p* < 0.01, indicating that real news articles elicited higher levels of death-thought accessibility than fake news. Next, results showed that death-thought accessibility was negatively associated with the number of death-related words recalled, *B* = − 0.05, *p* < 0.01, indicating that participants who reported higher levels of death-thought accessibility recalled lesser death-related words. Finally, the results revealed an indirect effect of news type on the number of death-related words recalled, *B* = 0.03, 95% *CI* = [0.001, 0.052]. Thus, death-thought accessibility also mediated the relation between news type and attention to death-related cues.

#### News-sharing intention

An independent samples t-test was conducted to examine if participants intend to share fake news articles more than real news articles. Results revealed that participants did not differ in their intent to share news articles between the fake news condition (*M* = 2.61, *SD* = 1.26) and the real news condition (*M* = 2.37, *SD* = 1.37), *t*(193) = − 1.30, *p* = 0.19.^1^

To examine if death-related thoughts spurred news-sharing intention, we employed a mediation analysis using Hayes’ PROCESS model 4 (Hayes, 2018) where news article type (real news was coded as 0, fake news was coded as 1) was included as the independent variable, news-sharing intention was included as the dependent variable, and death-thought accessibility was included as the mediator. Consistent with our previous findings, results showed that news type was negatively associated with death-thought accessibility, *B* = − 0.49, *p* < 0.01, where real news articles elicited higher levels of death-thought accessibility. Next, results also revealed that death-thought accessibility was positively associated with news-sharing intention, *B* = 0.45, *p* < 0.01. Individuals who reported experiencing higher levels of death-thought accessibility indicated that they were more likely to engage in news-sharing. Finally, results indicated that news type was negatively associated with news-sharing intention via death-thought accessibility*, B* = − 0.22, 95% *CI* = [− 0.39, − 0.07],^2^ thus demonstrating the mediation effect of death-thought accessibility on the relation between news type (fake versus real news) and news-sharing intention.

Similar to Study 1, we conducted extra tests to examine the influence demographic characteristics may have on the results reported above. The results of these tests showed that even when demographic characteristics were accounted for, the critical results remained—death-thought accessibility was higher for real news than fake news, attention to death-related cues and news-sharing intentions did not differ across the two conditions. However, it is to note that the initial significant effect of news type on negative affect was no longer observed, and a marginal significant effect of news type was found for positive affect after accounting for the demographic characteristics.

### Discussion

Our findings in Study 2 replicated the finding in Study 1 that real news increased death-thought accessibility more than fake news. Although we did not find a direct effect of news type on news-sharing intention, we found that death-thought accessibility, which was aroused from the real news articles, led to an increased intention of sharing the news articles. This suggests that it is not news type per se (i.e., fake news or real news) that influenced one’s intent to share the news they have read, but rather, it was the increased death-thought accessibility from reading the news that encouraged the behavior. In other words, one would be inclined to share news articles as long as death-thought accessibility was increased, regardless of whether the article was real or fake news.

Finally, participants who read the real news articles, which elicited more death-related thoughts, recalled *fewer* death-related words, compared to participants who read the fake news articles. This suggests that increased death-thought accessibility was associated with *decreased* attention to death-related cues. According to the TMT, existential anxiety produced by death-related thoughts is managed differently depending on whether death-related thoughts were conscious or not (Pyszczynski et al., 1999). When death-related thoughts are conscious (in focal attention), proximal defences are engaged, and existential anxiety is managed either by suppressing death-related thoughts or convincing oneself that death is not an immediate problem. In contrast, when death-related thoughts are out of consciousness (not in focal attention), distal defences are activated; in this case, existential anxiety is dealt with in a more indirect symbolic manner focused on one’s worldviews, self-esteem, and close relationships. Conscious death-related thoughts may also trigger distal defences, but only after delay and distraction (Greenberg et al., 1994). As such, the negative relationship found between death-thought accessibility and the attention to death-related cues could be due to proximal defenses being engaged. In other words, the accessibility to death-related thoughts may have been conscious, and participants may have attempted to suppress those thoughts, which explains the lower recall of death-related words by participants who reported higher levels of death-thoughts accessibility.

## Study 3

In Study 1 and 2, the effects of fake news and real news were examined by having participants read through a series of 8 Facebook posts featuring content from fake news articles or real news articles. This introduces a potential confound to the effects of news type (fake versus real) on the variables we have examined thus far—the content of the news articles. The contents within the set of the 8 fake news articles were different from the set of the 8 real news articles. As such, it is likely that our findings from Study 1 and 2 were driven by the *content* of the news articles rather than the *type* of news article and its accompanying characteristics (i.e., fake versus real). To manage this, Study 3 employed news materials that were matched in content; we developed a fake news article based on the content from a real news article.

### Participants

Given the effect sizes that were found in Study 2, we conducted an a-priori power analysis and calculated the minimum sample size required to detect a medium effect size of *d* = 0.50 for this study. A-priori power analysis by G-Power (Faul & Erdfelder, 1992) indicated that a minimum sample size of *n* = 128 (*n* = 64 per condition) is required to detect such an effect. A total of 188 responses were collected through Amazon’s Mechanical Turk. However, as 64 participants did not pass an attention check (describe in the later section), a final sample size of 124 participants was used in the subsequent analyses (73 females, *M*_age_ = 39.48, *SD*_age_ = 13.20). All participants resided in the USA; most of them were Americans (114 Americans) and identified “Caucasian” as their ethnic identity (91 Caucasians, 8 African Americans, 8 Hispanics, 7 Asians, 9 Native Americans, 1 N/A). All but 4 participants indicated that their native language was English. The majority of the participants had at least a college degree (75 College degrees, 22 Masters, 3 PhDs, 24 High School and below).

### Design and procedure

Methods were identical to those of Study 1 and 2 except where noted below. Participants were randomly assigned to one of two conditions in an in-between participants design (*N*_fake news_ = 62, *N*_real news_ = 62). After participants were shown the Facebook post, they responded to an attention check that asked what the content of the post was on. This was then followed by the full news article. Participants who failed the attention check were not allowed to proceed with the study. Those who have passed the attention check proceeded with indicating how they felt and responding to items that measured death-thought accessibility. After which, they completed a 20-question-word pair task, indicated their news-sharing intentions, and provided their demographic information before completing the study.

### Materials

#### News articles

Unlike Study 1 and 2, participants were shown one Facebook post (consisting of a title and a short summary) and the full news article of the Facebook post. Participants in the fake news condition read one Facebook post and its article, which contained a piece of fake news, while participants in the real news condition read one Facebook post and its article, which contained a piece of real news. To keep the content of the news consistent across both fake news and real news conditions, we recruited the help of a media expert, an online news journalist, to create a piece of fake news article based on a real news article. The news article was sourced from a professional news website (i.e., AP News) and was selected based on the relatedness of the news article—the selected article concerns the COVID-19 vaccine (McNeil, 2020). In developing the fake news article, the online news journalist edited the content of the news article by changing the headlines, omitting correct information, and adding false information. By having a fake news article and a real news article based on the same content, we were able to tease apart the effect of news type (i.e., the characteristics of fake versus real news) from the effect of its content. The news articles are included in the Additional file 1: Appendix.

#### Affect

Negative affect and positive affect were measured using the same items from Study 1 and 2. The items were averaged to form a single index for negative affect (*M* = 2.16, *SD* = 1.06, α = 0.95) and positive affect (*M* = 3.10, *SD* = 1.01, α = 0.92).

#### Death-thought accessibility

Death-thought accessibility was measured using the same items from the previous studies. The two items were significantly correlated (*r* = 0.72), and hence were combined to form a single index for death-thought accessibility (*M* = 2.39, *SD* = 1.22).

#### Attention to death-related cues

Attentiveness to death-related cues was assessed through the question-word pair task used in Study 2. The number of death-related words they recalled served as an index for their attention to death-related cues (*M* = 1.53, *SD* = 1.63).

#### News-sharing intention

News-sharing intent was measured with the same items from Study 2. The set of three items adopted from Lee and Ma (2013) correlated with the single item measure we included for this study (*r* = 0.84). The items were averaged to form a single index for news-sharing intent (*M* = 2.94, *SD* = 1.30, α = 0.95).

### Results

#### Affect

Independent samples t-tests were conducted to examine the mean differences in negative affect and positive affect elicited by the fake news article versus the real news article. Results revealed that there were no significant differences in negative affect (*p* = 0.34) and positive affect (*p* = 0.25) between the fake news and the real news conditions.

#### Death-thought accessibility

An independent samples t-test was conducted to examine if real news articles increased death-thought accessibility more than fake news articles, as we have observed in the previous two studies. Although results showed that death-thought accessibility was higher in the real news condition (*M* = 2.51, *SD* = 1.23) than the fake news condition (*M* = 2.27, *SD* = 1.22), the difference in death-thought accessibility between the two conditions was not significant in this study, *t*(122) = 1.10, *p* = 0.27.

#### Attention to death-related cues

Similar to Study 2, given the positive skew observed in the recall of death-related words (skewness = 2.02), the data were log-transformed for the analysis. An independent samples t-test was conducted to examine if attention to death-related cues differed across the real and fake news conditions. Results revealed that participants in both real news and fake news conditions recalled a similar number of death-related words (*p* = 0.62).

#### News-sharing intention

An independent samples t-test was conducted to examine if news-sharing intention differed between the fake news group and the real news group. Results revealed that there was no significant between the two conditions for news-sharing intention (*p* = 0.71).^3^

Given that there was no significant effect of news type of death-thought accessibility, we did not proceed to test a mediation for attention to death-related cues and news-sharing intention as we did in Study 2. However, a simple linear regression analysis revealed that death-related thoughts predicted news-sharing intention (*b* = 0.42, *t*(123) = 4.69, *p* < 0.001).^4^ This is observed in both the real news condition (*b* = 0.33, *t*(61) = 2.53, *p* = 0.014) and the fake news condition (*b* = 0.51, *t*(61) = 4.10, *p* < 0.001). Death-related thoughts also predicted attention to death-related cues (log-transformed) (*b* =− 0.05, *t*(106) =  − 2.25, *p* = 0.03). However, this was only observed in the real news condition (*b* = − 0.12, *t*(50) =  − 4.74, *p* = 0.014), but not the fake news condition (*b* = 0.302, *t*(55) = 0.59, *p* = 0.56).

Similar to the previous studies, we conducted extra tests to check for the influence demographic characteristics may have on the results reported above. Results indicated that the reported findings remained—no significance difference was found for death-thought accessibility, negative affect, positive affect, attention to death-related cues and news-sharing intentions across the real news and fake news condition.

### Discussion

The findings of Study 3 demonstrated that participants did not respond differently toward the fake news article and the real news article; both types of articles produced similar levels of negative affect, positive affect, death-thought accessibility, attention to death-related cues and news-sharing intention. These results suggest that when news content was controlled for, people did not react differently to fake news and real news. In other words, the characteristics of fake news articles (such as exaggerating, omitting or adding false information while maintaining the news format) did not exert a significant influence over affective responses, attention and news-sharing intent. These results also demonstrate that the content of news articles is an important factor in influencing death-thought accessibility and news-sharing intention. With fake news containing more death-related themes (Molina et al., 2019), then theoretically, it will be likely that one’s accessibility to death-related thoughts is higher for fake news than real news. It is likely that we found higher levels of death-thought accessibility in the real news than the fake news condition in Studies 1 and 2 because there were more death-related themes in the articles included in the real news condition than the fake news condition.

Death-thought accessibility was found to predict news-sharing intention, which was consistent with the findings of Study 2. This supports the idea that sharing news articles is a way for people to resolve the existential anxiety they experience when they come across news that had increased their accessibility to death-related thoughts. Death-thought accessibility was also found to predict attention to death-related cues, which was also consistent with what we found in Study 2. Given that a negative relation between death-thought accessibility and the attention to death-related cues was also found in this study, it provides further support to the likelihood that proximal defenses were engaged. The lower recall of death-related words by participants who reported higher levels of death-thoughts accessibility is likely to reflect their attempts in suppressing conscious death-related thoughts. However, this negative relation between death-thought accessibility and attention to death-related cues is apparent only in the real news condition and not the fake news condition, which suggests that attempts to suppress conscious death-related thoughts were made upon reading the real news article and not the fake news article. Given that death-thought accessibility was not significantly higher in the real news condition compared to the fake news condition, it is probable that people’s prior knowledge of the news article introduced other motivations, such as countering fake news (Are, 2019; Lewandowsky & van der Linden, 2021; Pulido et al., 2020), that could have influenced the attention they pay to death-related cues. If participants in the fake news condition knew that the news article they read was fake, it is likely that they would be motivated to identify cues characteristic of fake news instead of ignoring death-related cues to suppress death-related thoughts. Moreover, being motivated to counter fake news may also serve to explain why death-thought accessibility still predicted news-sharing intention in the fake news condition—people were motivated to share that the news article to others so that more people would know that the news article is fake (Are, 2019; Pulido et al., 2020).

## General discussion

This paper sought to provide an explanation for *why* people tend to share fake news over real news, and we proposed that the increased accessibility to death-related thoughts is the unconscious driving force behind news-sharing. In Studies 1 and 2, we showed that fake news *did not* increase accessibility to death-related thoughts and negative affect, but instead, real news did. Furthermore, we showed that increased accessibility to death-related thoughts was not only associated with decreased attention to death-related cues, but more critically, it was associated with an increased inclination to share news articles. In Study 3, when we controlled for the content in a fake news article and a real news article, we found no differences in death-thought accessibility and news-sharing intention between the two conditions; however, death-thought accessibility continued to predict attention to death-related cues and news-sharing intention. Overall, our findings suggest that it is not news type per se (i.e., real news versus fake news) that motivates news-sharing, but rather, it is the increased death-thought accessibility from reading news articles that encourages news-sharing.

Our findings were contrary to what we predicted; real news articles turned out to increase death-thoughts accessibility and negative affect more than fake news, even though studies have shown that fake news tends to contain death-related themes (Molina et al., 2019) and elicits negative emotions (Vosoughi et al., 2018). However, with no such differences found in Study 3, it is likely that these results was an artifact of the materials used in Studies 1 and 2. The content included in the real news condition probably contained more death-related themes than those included in the fake news condition.

Across the studies, we found that the increased accessibility to death-related thoughts was associated with an increased tendency to share news-articles, which is consistent with the TMT framework (Greenberg et al., 1986; Pyszczynski et al., 2015). In line with this theoretical framework, being aware of death led to behaviors that were aimed at resolving existential anxiety, that includes seeking closeness to others (Bowlby, 1969, 1982; Mikulincer et al., 2003; Pyszczynski et al., 2004; Solomon et al., 1991). Social media platforms are designed to encourage sociality that motivates interaction with their close family and friends (Alaimo & Kallinikos, 2019). The sharing of news on social media harnesses a sense of connectedness and belonging (Quinn & Oldmeadow, 2013; Ryan et al., 2017; van Eldik et al., 2019). As such, given that sharing news articles allows one to feel connected to others and provides a sense of belonging (Quinn & Oldmeadow, 2013; Ryan et al., 2017; van Eldik et al., 2019), the increased inclination for people to share news upon the increased accessibility to death-related thought supports the TMT framework. It is also worth noting what our news-sharing measure reflects: people are not only inclined to share the specific news articles they have read, but they are also inclined to share new-articles on Facebook more generally upon the increase in death-thought accessibility. Moreover, given that the effect of news type on news-sharing intention is present *only* after accounting for increased death-thought accessibility, the current work provides evidence for an underlying explanation for news-sharing inclinations. This research contributes to the existing literature where research on fake news primarily focuses on the manner in which fake news spreads (Mustafaraj & Metaxas, 2017; Shin et al., 2018) and the literary style of fake news (Horne & Adali, 2017; Vosoughi et al., 2018). Furthermore, our work adds to the literature on media psychology that has employed the TMT framework (e.g., Fischer-Preßler et al., 2019; Pyszczynski et al., 2003), evidencing its applicability in explaining people’s responses to media.

## Limitations and future directions

Our work is far from conclusive and poses questions for future work. Within the TMT framework, existential anxiety that is produced by increased death-thought accessibility is dealt with differently, depending on the consciousness of the thought (Pyszczynski et al., 1999). Conscious death-related thoughts activate proximal defenses, which involves the use of rationing thinking, such as suppressing death-related thoughts or convincing oneself that death is not an immediate problem. Non-conscious death-related thoughts, in contrast, activates distal defenses, which involves a more indirect and symbolic approach focused on worldviews, self-esteem and close relationships. As we did not measure the consciousness of death-related thoughts evoked by the stimuli in our studies, it is not possible for us to identify the specific defense that was engaged. On the one hand, the decreased attention to death-related cues with increased death-thought accessibility suggests that proximal defenses were engaged—participants may be suppressing death-related thoughts during the task. On the other hand, the increased tendency to share news articles with increased death-thought accessibility suggests that distal defenses were activated as participants sought to maintain close relationships by sharing the news articles. It is also likely that both defenses are engaged during the studies as distal defenses can be engaged even with conscious death-related thoughts, but only after delay or distraction (Greenberg et al., 1994). Without knowing the level of consciousness of death-related thoughts that were elicited by the news article, we cannot be sure if the results were natural responses or reactive responses (i.e., purposeful suppression) to the stimuli. Taking our findings to the higher levels of death-thought accessibility by real news than fake news as an example, we are not able to elicit further if real news indeed elicited more death-related thoughts or if participants were suppressing their responses toward fake news. As such, it is ideal for future research to measure the consciousness of death-related thoughts elicited from news articles in order to reach definite conclusions.

There may also be other motivations behind news-sharing that should be considered, especially the extent to which people are motivated to counter fake news. News coverage of negative events like terrorist acts, high-profile crimes (Hameleers et al., 2020; Innes et al., 2019; Wang & Zhuang, 2018), and news on public health hazards like COVID-19 (World Health Organization, 2020) can trigger a spike in the volume and speed of fake news stories, misinformation, and opinion-based articles. With continued public concerns regarding the circulation of fake news online, individuals may be motivated to counter fake news by sharing real news articles. Evidence-based truthful information has been produced by various authorities and groups to counter fake news (Are, 2019; Pulido et al., 2020). Individuals may also share fake news articles to create awareness of misinformation as a means of countering fake news. As such, these motivations would conflate with the responses organically produced from news articles and exert an influence over news-sharing intentions. Hence, it is important for future research to consider such motivations in order to tease apart the effects of such motivations from the effect of news type (fake versus real) on news-sharing behavior.

It is also important to underline that our study focused on news-sharing *intentions* as opposed to news-sharing *behavior*. According to the theory of planned behavior (Ajzen, 2002), the ultimate decision to act depends on factors beyond intention alone; this includes their attitudes toward sharing, the norms of sharing, and the perceived control of sharing (Ajzen, 2002). Our findings showed that increased death-thought accessibility led to greater *inclinations* to share news articles. To conclude that death-thought accessibility influences news-sharing *behaviors,* it is necessary for future research to consider these other factors that lead up to behavior or to measure behavior itself.

We recognize that participants in both studies came from WEIRD populations (see Henrich et al., 2010). Given the far-reaching capabilities of social media platforms, where most people consume the news in modern times (Shearer, 2018), it is likely that the results can generalize to other modern societies where social media is integral to the everyday lives of people.

The evidence from the present study, while preliminary, have important theoretical and practical implications. To date, researchers have uncovered several aspects of fake news; most were focused on correcting the effects of fake news (e.g., De Keersmaecker & Roets, 2017; Nyhan & Reifler, 2010; Thorson, 2016), and the cognitive processes that underlie the extent to which individuals believe fake news to be true (De Keersmaecker & Roets, 2017; Ipsos, 2018; Lazer et al., 2018; Pennycook & Rand, 2019; Silverman & Singer-Vine, 2016; Spohr, 2017). All these studies hint at the prevention against the effects of fake news after its exposure. The present research extends this literature by focusing on the process before fake news is spread to a wider audience. By identifying death-related thoughts to be the underlying factor behind news-sharing, the present research can inform researchers, practitioners (e.g., media outlet, fact-checkers) and regulatory bodies (e.g., government agencies) in designing effective interventions to stifle the spread of fake news. For instance, rather than sharing news articles to promote close relationships, potential interventions can consider reducing death-thought accessibility and resolving existential anxiety by validating one’s worldviews or by enhancing one’s self-esteem. With the TMT framework, our current findings can provide important understandings of management fake news following events, such as disasters (Chen & Sakamoto, 2013), terror attacks (Fischer-Preßler et al., 2019) or most relevant in the current state of affair, a global pandemic (Brainard & Hunter, 2020; Pyszczynski et al., 2020).

## Conclusion

Fake news has real consequences as people become confused and misunderstand information. Yet, studies examining the spread of fake news have largely focused on how fake news is shared and the literary style of fake news. Little is known about how characteristics of fake news interact with our psychology to motivate news-sharing. Drawing on the Terror Management Theory, we offer an explanation to why certain news articles are shared more. Across three experimental studies, we found that it was not news type per se (i.e., fake news or real news) that influenced news-sharing inclinations; rather, it was death-related thoughts elicited from the content of news articles that motivated news-sharing. Collectively, our results support the Terror Management Theory and, more importantly, elucidate the underlying reason behind news-sharing behaviors.

## Supplementary Information

**Additional file 1**. Appendix 1.

## Data Availability

The materials and datasets used and analyzed during the current study are available from the corresponding author on reasonable request. The experiments was not preregistered.

## Abbreviations

COVID-19: Coronavirus disease 2019
TMT: Terror management theory
PANAS: Positive and Negative Affect Schedule
WEIRD: Western, educated, industrialized, rich and democratic

## Footnotes

1 A separate independent samples t-test was also conducted to test if there was a difference in the intention to share the specific series of Facebook posts they have read across the two conditions. Again, results revealed that participants did not differ in sharing intentions of the news articles they have read across the fake news condition and the real news condition (*p* = .96).

2 We conducted another similar mediation analysis using Hayes’ PROCESS model 4 (Hayes, 2018), except this time, we included the item that specifically measured participants’ tendency to share the Facebook posts they have read in the study as the dependent variable. Results revealed a similar finding as the above. News type was negatively associated with death-thought accessibility, *B* = − 0.49, *p* < .01, where real news articles elicited higher levels of death-thought accessibility. Additionally, death-thought accessibility was positively associated with news-sharing intentions, *B* = 0.45, *p* < .01, indicating that higher levels of death-thought accessibility indicated that they were more likely to engage in news-sharing. Finally, results indicated that news type was negatively associated with news-sharing intention via death-thought accessibility*, B* = − 0.22, 95% *CI* = [− 0.39, − 0.08].

3 News-sharing intention specific to the news article they have read in the study also did not differ across the two conditions (*p* = .33).

4 Simple linear regression also revealed that death-related thoughts predicted the specific sharing intention of the article they read for the study (*b* = .46, *t*(123) = 4.57, *p* < .001).

## Abbreviations

COVID-19: Coronavirus Disease 2019
TMT: Terror Management Theory
PANAS: Positive and Negative Affect Schedule
WEIRD: Western, educated, industrialized, rich and democratic
