# Supplementary material for: Infodemic: the effect of death-related thoughts on news-sharing
Source: Cogn Res Princ Implic. 2021 May 20;6:39. doi: 10.1186/s41235-021-00306-0 (PMC8136755; doi:10.1186/s41235-021-00306-0)
Supplement: Supplementary file 1 — Additional file 1. Appendix 1. [file 41235_2021_306_MOESM1_ESM.docx]

**Appendix 1**

**News articles used in Study 1 and Study 2**

*Facebook posts based on real news.*


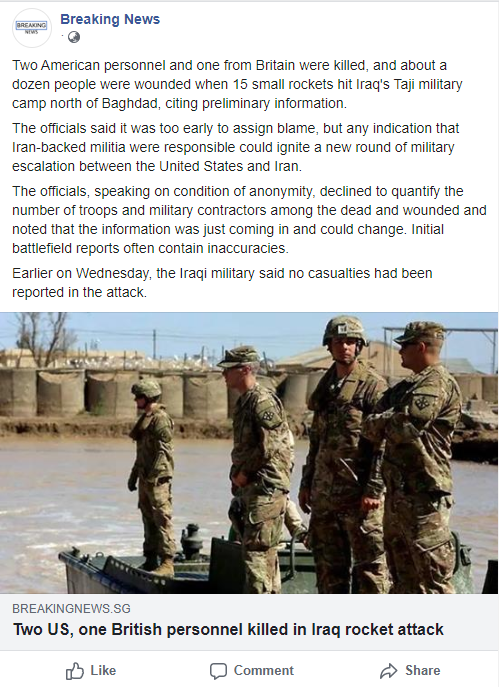

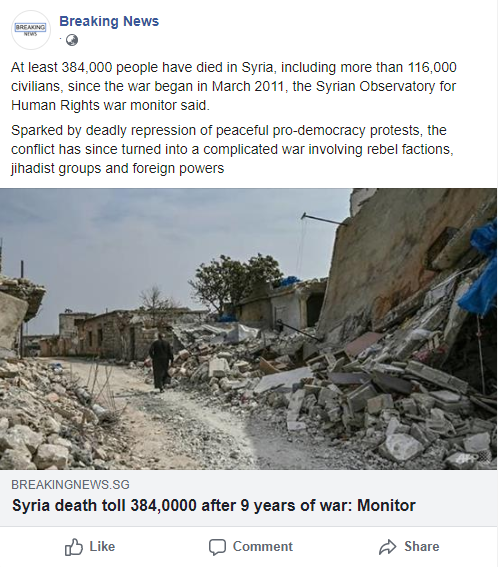

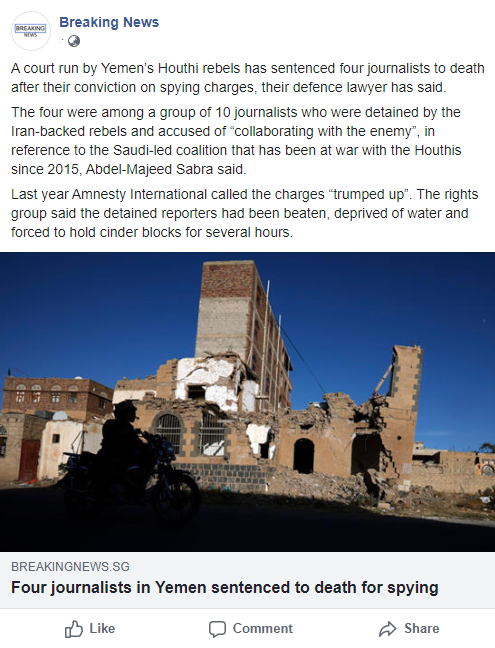

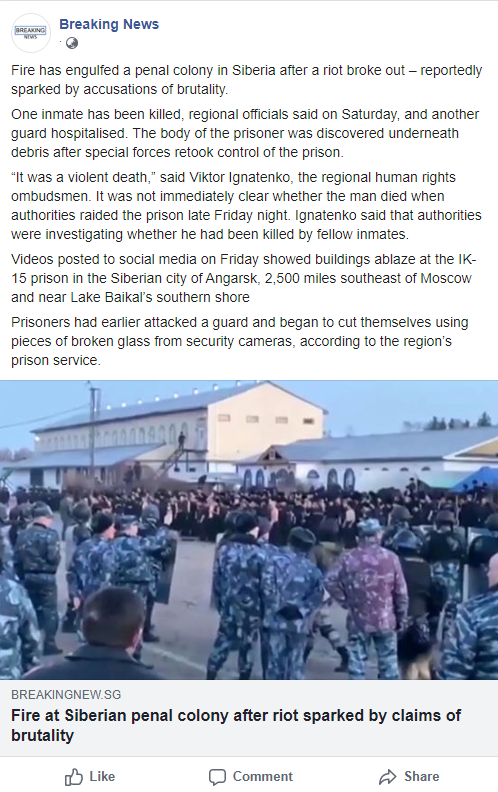


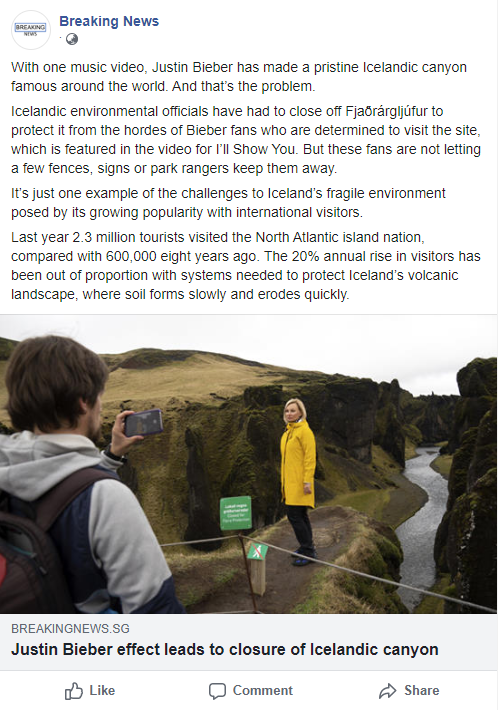

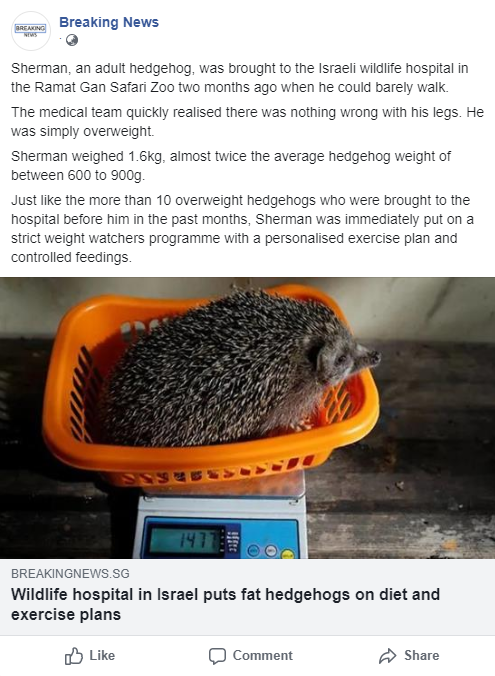

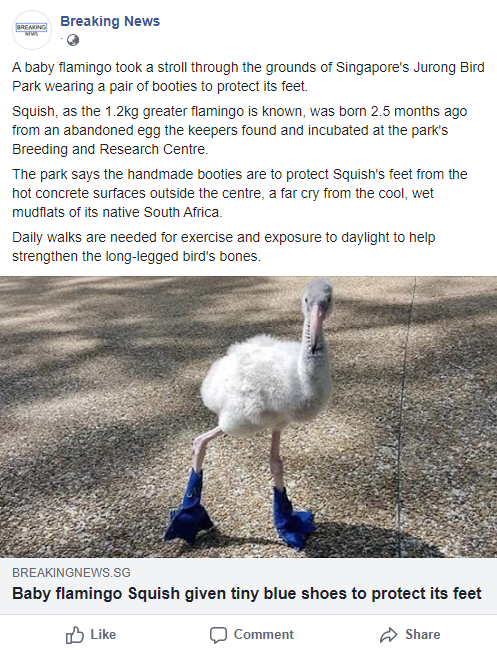

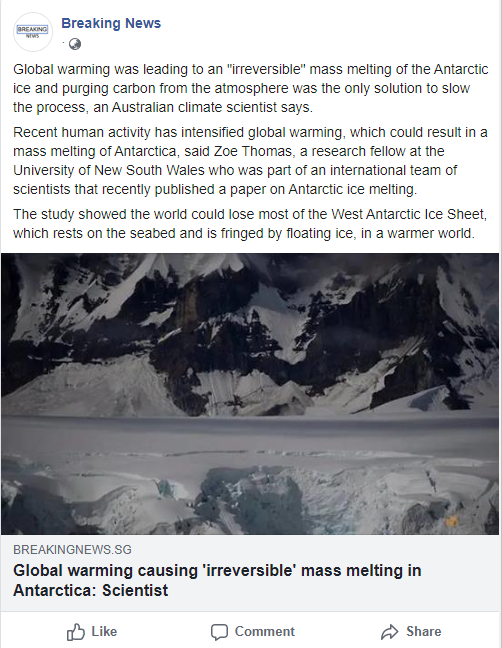


*Facebook posts based on fake news.*

**
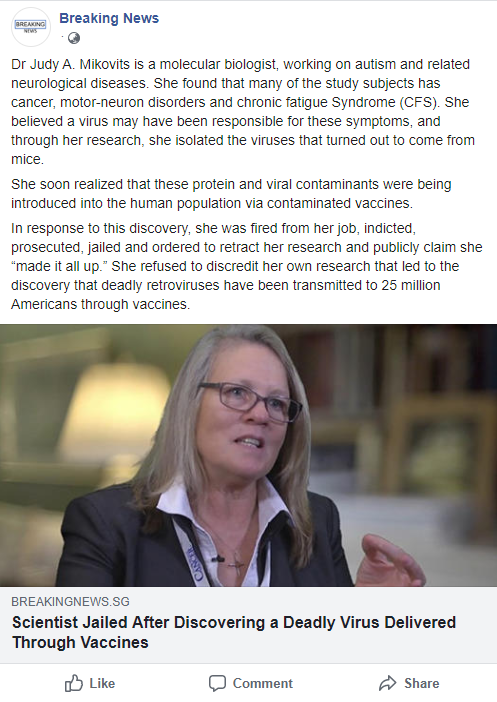
** **
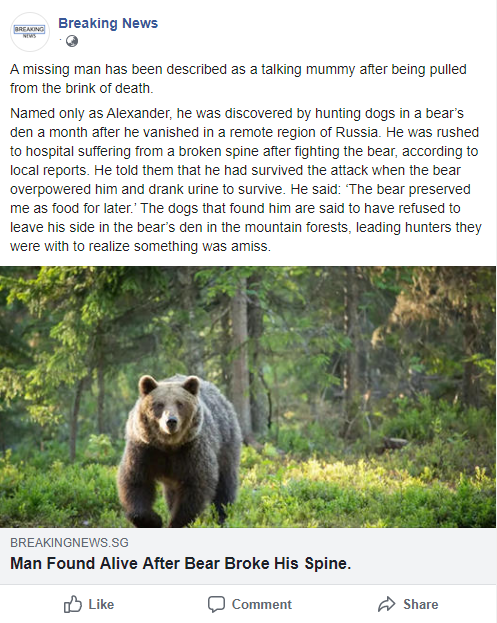

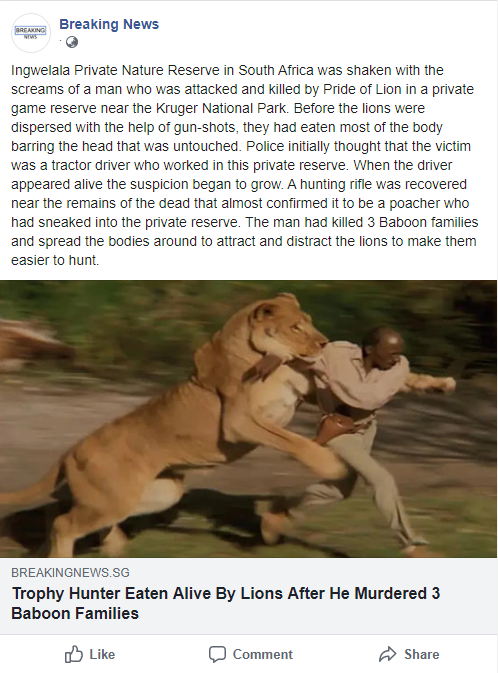
** **
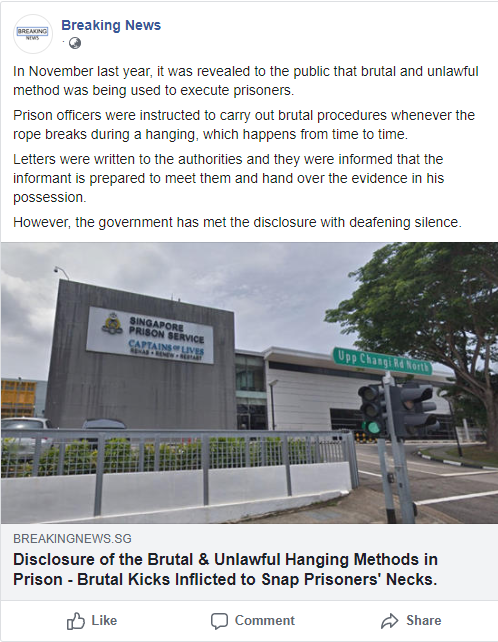
**
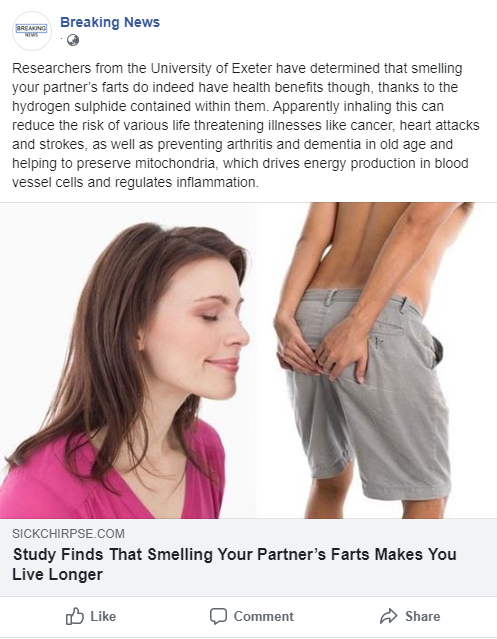

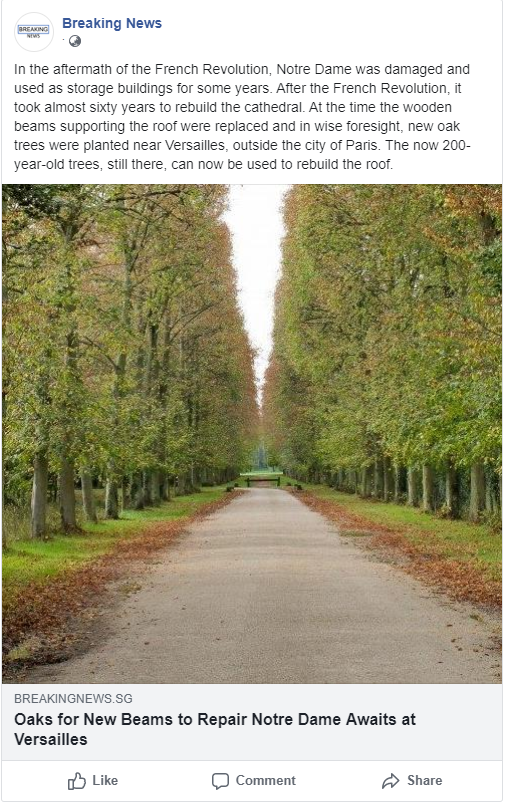

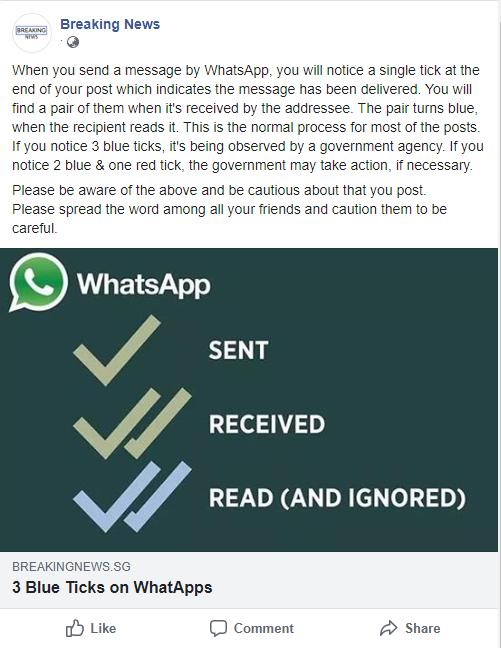

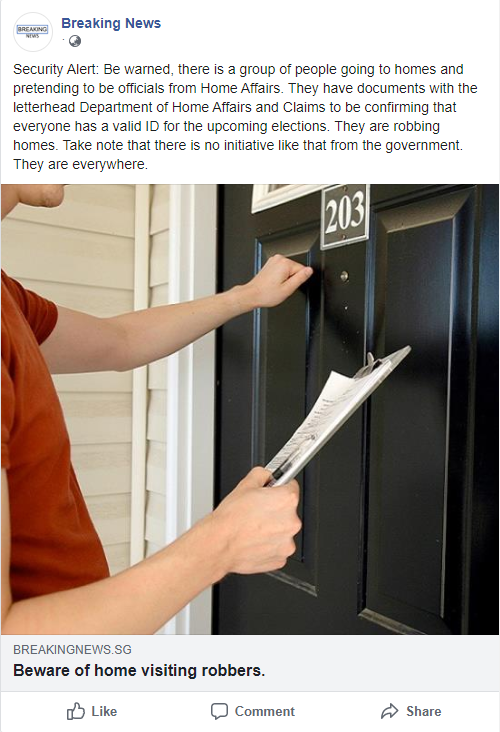


**News articles used in Study 3**

*Real news Facebook post.*

*
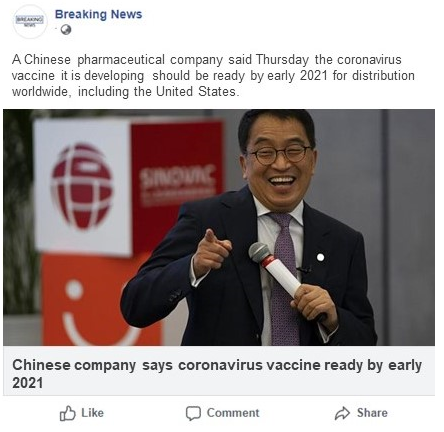
*

*Real news full article.*

Source: <https://apnews.com/article/virus-outbreak-beijing-china-europe-archive-0d82f2fce1c68560278d973c9cdc1e97>

A Chinese pharmaceutical company said Thursday the coronavirus vaccine it is developing should be ready by early 2021 for distribution worldwide, including the United States.

Yin Weidong, the CEO of SinoVac, vowed to apply to the U.S. Food and Drug Administration to sell CoronaVac in the United States if it passes its third and final round of testing in humans. Yin said he personally has been given the experimental vaccine.

“At the very beginning, our strategy was designed for China and for Wuhan. Soon after that in June and July we adjusted our strategy, that is to face the world,” Yin said, referring to the Chinese city were the virus first emerged.

“Our goal is to provide the vaccine to the world including the U.S., EU and others,” Yin said.

Stringent regulations in the U.S., European Union, Japan and Australia have historically blocked the sale of Chinese vaccines. But Yin said that could change.

SinoVac is developing one of China’s top four vaccine candidates along with state-owned SinoPharm, which has two in development, and military-affiliated private firm CanSino.

More than 24,000 people are participating in clinical trials of CoronaVac in Brazil, Turkey, and Indonesia, with additional trials scheduled for Bangladesh and possibly Chile, Yin said. SinoVac chose those countries because they all had serious outbreaks, large populations and limited research and development capacity, he said.

He spoke to reporters during a tour of a SinoVac plant south of Beijing. Built in a few months from scratch, the plant is designed to enable SinoVac to produce half a million vaccine doses a year. The bio-secure facility was already busy on Thursday filling tiny bottles with the vaccine and boxing them. The company projects it will be able to produce a few hundred million doses of the vaccine by February or March of next year.

SinoVac is also starting to test small doses of CoronaVac on children and the elderly in China after noticing rising numbers of cases globally among those two groups.

Yin said the company would prioritize distribution of the vaccine to countries hosting human trials of CoronaVac.

While the vaccine has not yet passed the phase 3 clinical trials, a globally accepted standard, SinoVac has already injected thousands of people in China under an emergency use provision.

Yin said he was one of the first to receive the experimental vaccine months ago along with researchers after phase one and two of human trials showed no serious adverse effects. He said that self-injecting showed his support for CoronaVac.

“This is kind of a tradition of our company,” Yin said, adding that he had done the same with a hepatitis vaccine under development.

Earlier this year, China permitted “emergency use” of vaccine candidates for at-risk populations like border personnel and medical workers if companies could show “good safety and good antibodies” from tests of about 1,000 people, Yin said.

SinoVac received that approval in June along with SinoPharm and CanSino, and was able to provide tens of thousands of doses of CoronaVac to Beijing’s municipal government, Yin said.

SinoVac employees qualified for emergency use of the vaccine because an outbreak inside the company would cripple its ability to develop a vaccine, he said. About 90% of the company’s staff have received it.

“We are confident that our research of the COVI-19 vaccines can meet the standards of the U.S. and EU countries,” Yin said.

*Fake news Facebook post.*

*
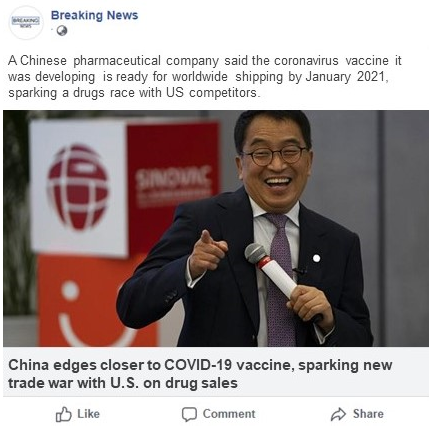
*

*Fake news full article.*

A Chinese pharmaceutical company said the coronavirus vaccine it was developing is ready for worldwide shipping by January 2021, sparking a drugs race with US competitors.

Yin Weidong, CEO of SinoVac, first announced human trials for the drug in April 2020, but encouraging signs of a 98% effectiveness rate with human test subjects has pushed forward plans for massive global delivery.

The company has revealed that it currently has five million doses ready to ship, with hundreds of millions more in production.

“This latest development is a testament to our commitment to combating COVID-19,” said Yin. “This is a massive leap forward, not just for China biotech, but the recovery of the global economy.”

SinoVac’s success currently puts them ahead of America’s leading developers of a vaccine.

A joint initiative by America’s Pfizer Inc and Germany’s BioNTech SE has developed a test vaccine that produces “more than 90% effectiveness” on human trials.

American drugmaker Moderna announced earlier last month that their vaccine had a 96% effectiveness rate, which placed it ahead of the pack until SinoVac’s announcement today.

SinoVac’s success sets China up for global dominance in what is now the most lucrative trade sector as nations around the world seek to emerge speedily from the pandemic.

Experts remain skeptical about a Chinese vaccine, given limited knowledge about the methodology and accuracy of human trials.

Stéphane Bancel, CEO of Moderna, expressed confidence that “what we have to offer is a safer and more robustly tested product”.

Stringent regulations in the U.S., Europe, Japan and Australia have historically blocked the sale of Chinese vaccines, but SinoVac’s latest announcement looks set to change that.

Countries like the Philippines, Thailand and Singapore have already placed substantive orders with the Chinese government for the first available vaccine.

SinoVac’s announcement is likely to push the U.S. to take preemptive steps to announce adoption of a U.S.-made vaccine in a bid to remain competitive in securing sales.

This is in spite of current uncertainty about the validity of China’s claims on human trials.

SinoVac’s has yet to pass globally accepted standards for drug trials, but the company has already injected hundreds of thousands of people in China under a government-mandated emergency use provision.

European intelligence sources have disclosed that these included forced trials with ethnic minority Uyghurs interned in Xinjiang prison camps.

Yin has dismissed these claims as “wild rumours sowed in malice”, asserting confidence in the company’s product.

He added that all SinoVac staff have already taken the vaccine to vouch for its reliability.

“We are confident that our research of the vaccines can meet the standards of the U.S. and EU countries,” Yin said.
